# Whole genome amplification approach reveals novel polyhydroxyalkanoate synthases (PhaCs) from Japan Trench and Nankai Trough seawater

**DOI:** 10.1186/s12866-014-0318-z

**Published:** 2014-12-24

**Authors:** Choon Pin Foong, Nyok-Sean Lau, Shigeru Deguchi, Takashi Toyofuku, Todd D Taylor, Kumar Sudesh, Minami Matsui

**Affiliations:** Synthetic Genomics Research Team, Biomass Engineering Program Cooperation Division, RIKEN Center for Sustainable Resource Science (CSRS), Yokohama, Kanagawa 230-0045 Japan; Ecobiomaterial Research Laboratory, School of Biological Sciences, Universiti Sains Malaysia, 11800 Gelugor Penang, Malaysia; Centre for Chemical Biology, Universiti Sains Malaysia, 11800 Gelugor Penang, Malaysia; R&D Center for Marine Biosciences, Japan Agency for Marine-Earth Science and Technology (JAMSTEC), 2-15 Natsushima-cho, Yokosuka, 237-0061 Japan; Laboratory for Integrated Bioinformatics, Core for Precise Measuring and Modeling, RIKEN Center for Integrative Medical Sciences (IMS), Yokohama, Kanagawa 230-0045 Japan

**Keywords:** Japan seawater, Polyhydroxyalkanoate (PHA), PHA synthase (PhaC), Whole genome amplification (WGA), Genome walking, *Marinobacter*

## Abstract

**Background:**

Special features of the Japanese ocean include its ranges of latitude and depth. This study is the first to examine the diversity of Class I and II PHA synthases (PhaC) in DNA samples from pelagic seawater taken from the Japan Trench and Nankai Trough from a range of depths from 24 m to 5373 m. PhaC is the key enzyme in microorganisms that determines the types of monomer units that are polymerized into polyhydroxyalkanoate (PHA) and thus affects the physicochemical properties of this thermoplastic polymer. Complete putative PhaC sequences were determined via genome walking, and the activities of newly discovered PhaCs were evaluated in a heterologous host.

**Results:**

A total of 76 putative *phaC* PCR fragments were amplified from the whole genome amplified seawater DNA. Of these 55 clones contained conserved PhaC domains and were classified into 20 genetic groups depending on their sequence similarity. Eleven genetic groups have undisclosed PhaC activity based on their distinct phylogenetic lineages from known PHA producers. Three complete DNA coding sequences were determined by IAN-PCR, and one PhaC was able to produce poly(3-hydroxybutyrate) in recombinant *Cupriavidus necator* PHBˉ4 (PHB-negative mutant).

**Conclusions:**

A new functional PhaC that has close identity to *Marinobacter* sp. was discovered in this study. Phylogenetic classification for all the *phaC* genes isolated from uncultured bacteria has revealed that seawater and other environmental resources harbor a great diversity of PhaCs with activities that have not yet been investigated. Functional evaluation of these *in silico*-based PhaCs via genome walking has provided new insights into the polymerizing ability of these enzymes.

**Electronic supplementary material:**

The online version of this article (doi:10.1186/s12866-014-0318-z) contains supplementary material, which is available to authorized users.

## Background

The Japanese ocean is special because of its ranges of latitude and depth. There are many deep-sea trenches and troughs with approximately 80% of the seafloor lying below 1000 m. The Japan Trench and Nankai Trough are part of the Pacific Ring of Fire where submarine volcanoes and hydrothermal vents are present along the tectonic plates. Two major sea surface currents, cold (Oyashio) from the North and warm (Kuroshio) from the South, flow along the Pacific coast of Japan and meet at the Japan Trench. These factors have contributed to the high diversity of marine species in the region [1]. Marine environments include some of the most diverse sets of microorganisms that have important roles in both biogeochemical cycles and ecosystems [2-4].

Polyhydroxyalkanoate (PHA) is naturally produced by many bacteria and some archaea under stressful or unbalanced nutritional conditions when there is an excessive supply of carbon sources [5-7]. This polymer can be used for bio-based and biodegradable plastic. Three major enzymes that are involved in the PHA biosynthetic pathway are β-ketothiolase (PhaA), NADP-dependent acetoacetyl-CoA reductase (PhaB) and polyhydroxyalkanoate synthase (PhaC). PhaC (EC 2.3.1-), the key enzyme involved in the polymerization process, determines the types of monomers (*R*-hydroxyacyl-CoAs) incorporated into the PHA polymer chain based on the enzyme’s substrate specificity [8-10].

PhaCs are categorized into four classes according to the subunits present and the substrate specificity [9]. Most of the PHA producers make Class I and II PhaCs. Primers targeting different classes of PhaC are available, e.g. general primers for Class I and II [11,12], Class II specific [13,14], Class III for *Haloarchaea* [15] and *Desulfococcus* [16], and Class IV for *Bacillus* spp. [17]. Although all the PhaCs share some common amino acid residues, it is not yet possible to amplify all four classes of PhaC with a single primer set. The general primers targeted to Class I and II PhaCs cover a wider range of bacterial genera (at least nine different genera) compared to the specific primer sets that target Class III and IV PhaCs.

To the best of our knowledge, this is the first attempt to identify PhaC diversity from seawater from different depths using a culture-independent approach. In this study, we decided to use the Class I and II general primers [12] to identify novel PhaCs from seawater. Seawater samples as deep as 3000 m and 5000 m were collected from the Nankai Trough and Japan Trench, respectively. This study also serves as the first trial using a whole genome amplification and genome walking approach [18] to determine the complete coding DNA sequence (CDS) of unstudied *phaC* genes followed by evaluation of PhaC activity for PHA production in *Cupriavidus necator *PHBˉ4 (a PHB-negative mutant strain).

## Results

### PHA synthase fragments from whole genome amplified seawater DNA

A total of 76 partial putative *phaC* clones were obtained from the seawater whole genome amplified (WGA) DNA. Twenty-one clones did not show any similarity with PhaCs in the GenBank protein sequences database using the BLASTX program and were excluded from subsequent analysis. The remaining 55 clones that contained the putative conserved domain superfamilies “PhaC_N” were classified into 20 different genetic groups (GGs) based on a cut off of 90% nucleotide sequence similarity. Seven clones containing an internal stop codon ‘TAG’ within the coding region of the putative PhaC were hypothesized to be pseudogenes. Three GGs (I-GG5, I-GG13 and II-GGb) were comprised solely of these pseudogenes.

Eighteen I-GGs and two II-GGs PhaCs were detected using the PhaC degenerate primer sets G-D and G-1R. These GGs were designated as “PhaC-I” or “PhaC-II” depending on the similarity with Class I or Class II PhaCs, respectively. This grouping generated similar results to the neighbor-joining phylogenetic tree (Additional file 1: Figure S2), where clones designated as the same “GG” were closely clustered in the same branch. For all the GGs, a putative lipase box (G-X-[S/C]-X-G) was identified from a MUSCLE alignment (Additional file 2: Figure S1). The only exceptions were I-GG12 and I-GG17 in which the first amino acid residue ‘glycine (G)’ in the lipase box motif was replaced by ‘serine (S)’ and ‘alanine (A)’, respectively. In addition, three amino acid residues, serine (S), aspartic acid (D) and tryptophan (W), that were conserved in the previously reported multiple sequence alignment of PhaC [9] were also present in all GGs.

**Figure 1:**
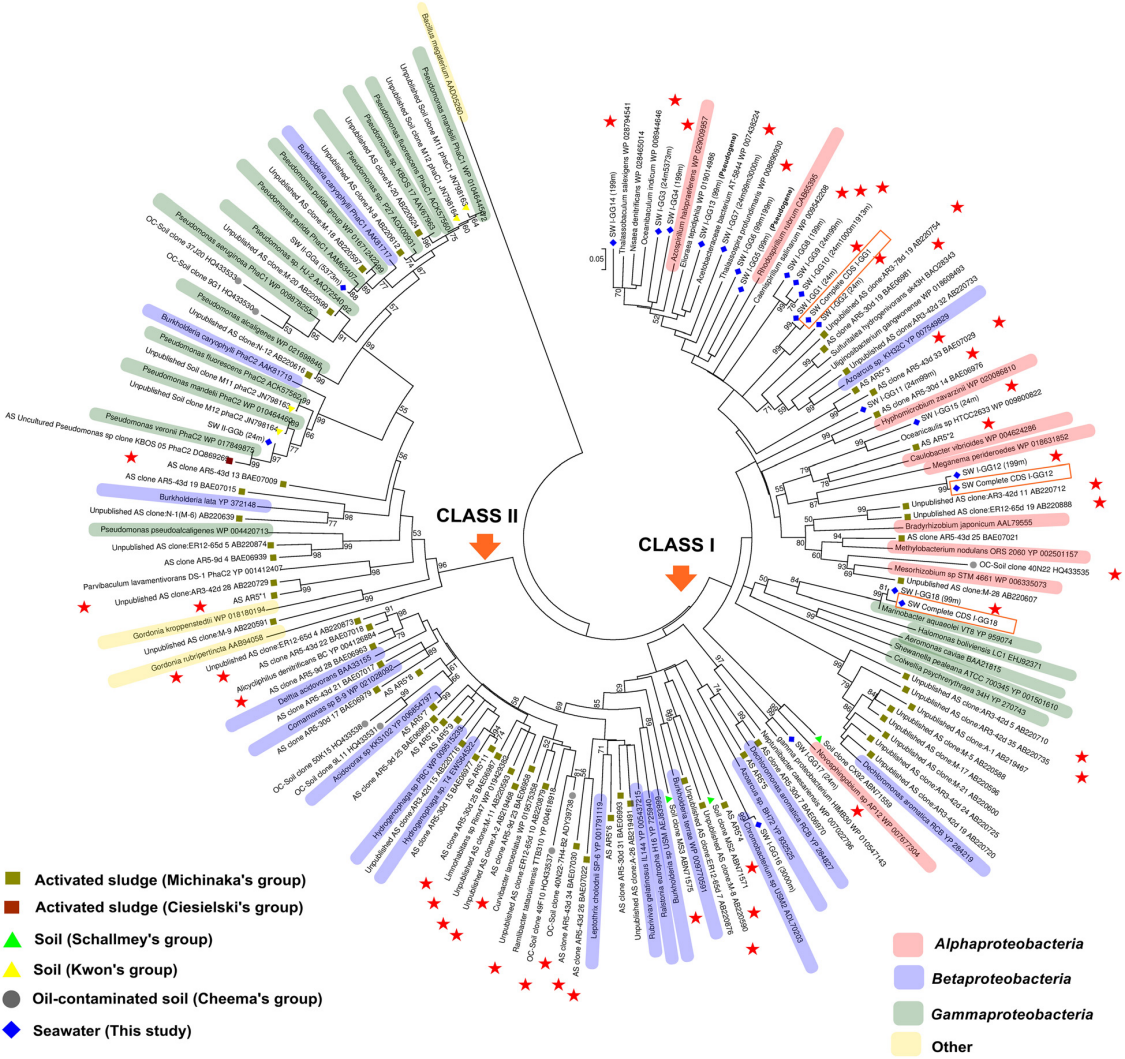
**Protein neighbor-joining phylogenetic tree of PHA synthases (PhaCs).** All putative PhaCs obtained from culture-independent studies, published [20-22,47] and unpublished were subjected to phylogenetic classification. Colored labels indicate the presence of known PHA producers in the same genus. Red stars indicate novel or uncharacterized putative PhaCs. Orange color boxes represent the complete CDS of PhaCs used for PHA production in this study. The scale represents the number of amino acid substitutions per site. Bootstrapping values less than 50 are not shown in the tree. Accession numbers for the PhaC sequences are indicated after the sequence name and can also be found in Additional file 4: Table S4. PhaC of *Bacillus megaterium* was used as an outgroup for the phylogenetic tree.

The protein sequence identities of 55 partial PhaC fragments against the GenBank protein sequences database ranged from 57% to 99% (Additional file 3: Table S3). There were five putative PhaC-GGs with very high identity matches (>90%): I-GG16, II-GGa and II-GGb to known PHA producers from the genera *Chromobacterium* (Class I) and *Pseudomonas* (Class II), and I-GG17 and I-GG18 to unknown PHA producers from *gamma proteobacterium* HIMB30 and genus *Marinobacter*, respectively (Figure 1).

Another four putative PhaC-GGs (I-GG5, I-GG13, I-GG14 and I-GG15) with BLASTX identity matches between 71-83% showed relatively close phylogenetic clustering with the PhaCs from the genera *Rhodospirillum, Acetobacteraceae bacterium, Oceanibaculum* and *Oceanicaulis*, respectively. The genus *Rhodospirillum* has a known PHA producer, *Rhodospirillum rubrum* [19], while the other genera include *phaC* genes annotated from the bacterial whole genome sequence (WGS). The remaining 11 putative PhaC-GGs (I-GG1 to GG4 and I-GG6 to GG12) that showed relatively distinct phylogenetic clusters when compared with the PhaC from cultured bacteria are believed to derive from yet uncultured bacteria.

### PhaC from culture-independent studies

Further comparison was carried out by compiling our *phaC* fragments and all predicted *phaC* fragments including unpublished data from various culture-independent studies (Figure 1) (Additional file 4: Table S4). Since our *phaC* fragments belonged to Class I and II, we only compared these two classes. In total, there are 301 putative *phaC* genes in the GenBank nucleotide sequences database (as of 22nd April 2014). Putative *phaC* genes with nucleotide sequence similarity >90% were assigned to the same genetic group.

The study on activated-sludge PhaC diversity [20] generated the largest number of partial *phaC* sequences with most of them belonging to Class I PhaC of *Betaproteobacteria*, followed by Class I PhaC of *Alphaproteobacteria* and Class II PhaC of *Gammaproteobacteria*. The same dominant group, Class I PhaC of *Betaproteobacteria*, was found in oil-contaminated soil DNA [21], followed by Class II PhaC of *Gammaproteobacteria* and Class I PhaC of *Alphaproteobacteria*. In contrast, Class I PhaC of *Alphaproteobacteria* was more dominant in the seawater DNA, followed by Class I PhaC of *Betaproteobacteria*, and Class I and II PhaC of *Gammaproteobacteria*. On the other hand, three complete CDSs of Class I PhaC of *Alphaproteobacteria* and *Betaproteobacteria* were isolated from the soil DNA genomic library [22]. Our seawater samples contained a higher proportion (15 out of 20 GGs) of novel or uncharacterized *phaC* fragments compared with samples from other environments.

### Isolation of complete coding region of *phaC* gene

**Figure 2:**
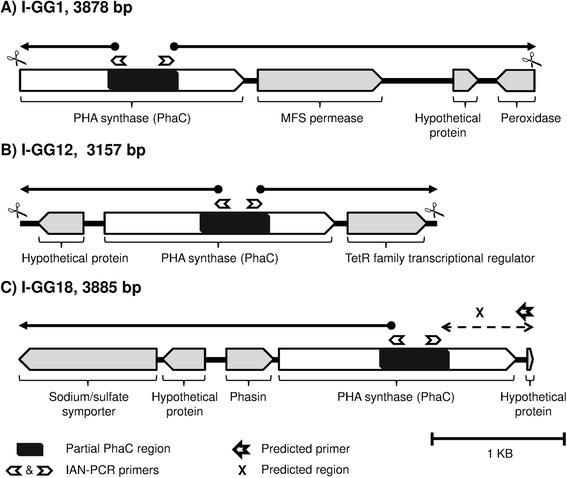
**Schematic diagram for the DNA fragments obtained by genome walking.** Major facilitator superfamily (MFS) permease is a membrane transporter. TetR is a tetracycline repressor protein. Phasin is a PHA granule-associated protein. The bold arrow lines indicate the DNA fragment amplified by IAN-PCR. The scissors indicate the restriction digestion site for *Eco*RI. For the DNA fragment I-GG18, the *Eco*RI digestion site could not be identified. The downstream region of the I-GG18 PHA synthase was determined via PCR amplification using a predicted primer based on the whole genome sequence (WGS) of *Marinobacter hydrocarbonoclasticus* strain ATCC 49840.

The complete coding regions of three Class I GGs were determined via *in silico* assembly of DNA fragments obtained by inverse affinity nested PCR (IAN-PCR) (Figure 2). These I-GGs were selected based on their low (I-GG12 = 57%), medium (I-GG1 = 68-73%) and high (I-GG18 = 99%) partial protein sequence identities with PhaC from the unknown PHA producers deposited in the GenBank protein sequences database (Additional file 3: Table S3). The length of the I-GG1 genome walking DNA fragment was 3878 bp. The downstream region showed close protein similarity with MFS permease (47% identity) followed by peroxidase (61% identity). The upstream region could not be determined due to the presence of an *Eco*RI restriction digestion site. This could be an incomplete CDS of a protein with approximately 8 to 14 amino acid residues missing at the N-terminal end based on multiple sequence alignment analysis (Additional file 5: Figure S3). The length of the I-GG12 genome walking DNA fragment was 3157 bp. The downstream region showed close protein similarity with the TetR family transcriptional regulator (58% identity). The length of the I-GG18 genome walking DNA fragment was 3885 bp (including the predicted region). We were only able to obtain the upstream region of the PhaC based on BLASTN similarity searches. Nonetheless, there was a high identity match (>95%) with PHA synthase and the upstream region (phasin and sodium/sulfate symporter) of *Marinobacter hydrocarbonoclasticus* ATCC 49840. Therefore, a reverse primer was designed based on the hypothetical protein located downstream of the PhaC of *Marinobacter hydrocarbonoclasticus* ATCC 49840.

The complete CDSs of I-GG1, I-GG12 and I-GG18 were confirmed by DNA sequencing and their closest PhaC matches from BLASTX analysis were *Oceanibaculum indicum* (65%), *Tistrella mobilis* (50%) and *Marinobacter sp.* C1S70 (97%), respectively (Table 1). High nucleotide sequence similarities (>96%) were observed between the partial *pha*C genes and their corresponding complete CDSs obtained by the genome walking method. The primary structure of the three putative complete PhaC CDSs indicated the presence of a lipase box motif. In addition, eight highly conserved amino acid residues were also present in these putative PhaCs (Additional file 5: Figure S3) based on observations reported in a previous study [23].

**Table 1:** **Closest organism matches of PHA synthase for the complete CDS** ***phaC*** **genes**

**Genetic group**	**Nucleotide length (bp)**	**Amino acid length (aa)**	**Closest organism match** ^**a**^	**Identity (%)**	**Accession no.**
**I-GG18**	1800	599	*Marinobacter* sp. C1S70	97	WP_022991790
**I-GG1**	1701	566	*Oceanibaculum indicum*	65	WP_008944646
**I-GG12**	1746	581	*Tistrella mobilis*	50	WP_014745461

^a^BLASTX against the GenBank non-redundant protein sequences database (nr), date: 21st September 2014.

### PHA polymerizing activity in *C. necator* PHBˉ4

The complete CDSs of three *phaC* sequences were subjected to functional analysis of PHA production in *C. necator* PHBˉ4, which is deficient in endogenous *phaC* function. Of the three *phaC*s, only I-GG18 was able to produce 60 wt% of P(3HB) (Table 2) under the control of the *phaC1* promoter from *Cupriavidus necator* H16. On the other hand, I-GG18 (without the promoter) did not show any PHA production under the same cultivation conditions.

**Table 2:** **PHA production of different** ***phaC*** **genes in recombinant** ***C. necator*** **PHBˉ4** ^**a**^

**Sample name**	**Dry cell weight (g/L)**	**PHA content (wt %)**	**PHA composition**
***C. necator*** **H16 (Wild type)**	4.1 ± 0.5	70 ± 5	P(3HB)
***C. necator*** **PHBˉ4(PHB-negative mutant)**	1.2 ± 0	n/d	n/d
**I-GG1**	0.9 ± 0	n/d	n/d
**I-GG12**	0.9 ± 0	n/d	n/d
**I-GG18**	2.6 ± 0.1	60 ± 8	P(3HB)
**I-GG18 (without promoter)**	0.9 ± 0	n/d	n/d

^a^Cells were cultivated in MM medium at 30°C, 200 rpm for 48 hours with 20 gL^−1^ of fructose as the sole carbon source.

n/d = not detected.

## Discussion

### Unexplored PHA synthases from the Japan Trench and Nankai Trough

The Japan Trench and Nankai Trough have unique environmental features, one of them is their seawater temperature which is influenced by surface currents and depth [1]. PHA producers have been isolated from various marine-associated environments [24,25], however, there are no previous reports on the study of PHA synthase from the Japan Trench and Nankai Trough. A degenerate primer set (G-D and G-1R) targeting Class I and II PhaCs developed by Romo *et al.* [12] was used in this study. We successfully isolated 76 partial *phaC* fragments and classified them into 20 GGs (including three PhaC-GGs comprised solely of pseudogenes) for both Class I and II PhaCs from whole genome amplified (WGA) seawater DNA.

In this study, 11 putative PhaC-GGs showed relatively low sequence identity (≤80%) and distinct phylogenetic lineage with the PhaCs from known PHA producers. We hypothesized that these PhaC-GGs are derived from yet uncultured bacteria. In addition, four putative PhaC-GGs that showed high sequence similarity (81-99%) with annotated PhaCs from unknown PHA producers (*Thalassobaculum salexigens*, *Oceanicaulis* sp., *gamma proteobacterium* HIMB30 and *Marinobacter* sp.). In total, 15 new or uncharacterized putative PhaC-GGs (excluding pseudogenes) were discovered from the WGA seawater DNA examined in this study.

Novel bacterial species have been discovered from the deep sea of both the Japan Trench and the Nankai Trough [26-28]. Indeed, the microbial biosphere in deep-sea environments is surprisingly diverse [2,29]. To date, the occurrence of PHA producers in deep-sea water that have been reported include *Moritella* sp. (8683 m) and *Shewanella* sp. (5110 m) from the Pacific Ocean [30], *Halomonas profundus* (2291 m) from the Mid Atlantic ridge [31] and *Pseudoaltermonas* sp. (1855 m) from Bohai Sea sediment [32]. Four sites located between the depths of 1000 m and 5373 m were sampled in this study. A total of five putative PhaC-GGs (I-GG3, I-GG7, I-GG10, I-GG16 and II-GGa) were identified from these deep-sea regions. Two putative PhaC-GGs obtained from 3000 m and 5373 m had very high sequence identity to known PHA producers from *Chromobacterium* sp. (98%) and *Pseudomonas* sp. (99%), respectively. We conclude that the occurrence of these clones was not due to experimental contamination because of the presence of non-random nucleotide mismatches. Most of these nucleotide mismatches occurred at third codon positions encoding for the same amino acids or functionally similar amino acid side groups. Neither of these genera has been reported previously to be deep-sea PHA producers. Nevertheless, several *Pseudomonas* sp. have been isolated from deep-sea environments [33-35], and isolates from the genus *Chromobacterium* have been found in the Southern Ocean below 200 m [36]. The remaining three putative PhaC-GGs were found to be conserved in water columns from different depths with clones detected in both shallow (24 m) and deep seawater (1913 m, 3000 m and 5373 m). We speculate that these three are derived from non-cultured bacteria due to their distinct phylogenetic lineage with known PHA producers.

We also attempted to examine the functionality of three uncharacterized putative PhaC-GGs from the WGA seawater DNA. Complete protein coding sequences are required for this purpose, thus a genome walking approach (IAN-PCR) was employed to obtain the complete CDSs. Of the three complete putative PhaC CDSs, only I-GG18 with high sequence identity to *Marinobacter* sp. showed 60 wt% of P(3HB) accumulation in recombinant *C. necator* PHBˉ4. The reason for the inability of recombinant *C. necator* PHBˉ4 harboring either I-GG1 or I-GG12 to produce PHA is unknown. The expression of the *phaC* gene alone may not form an active protein in the surrogate host and it may require some other factors to form an active enzyme [37]. Among the three genome walking DNA fragments, only I-GG18 had the *phaC* clustered with other PHA metabolism related genes, i.e., phasin (*phaP*), a PHA granule-associated protein. It is not unusual because it is known that in some bacteria the *phaC* is not present in the same operon with *phaA* and *phaB* [38].

To the best of our knowledge, this is the first *phaC* gene from the genus of *Marinobacter* that shows functional activity in a heterologous expression host. Bacteria from this genus have been discovered in various habitats and at all depths in the oceans [39]. Some species of bacteria from the genus *Marinobacter* have been shown to display the ability to degrade hydrocarbons [40-42] and PHA polymers [43]. Very recently, this genus was suspected to be one of the PHA producers [44], but so far no studies have been published confirming PHA production from pure cultures of *Marinobacter* sp. In this study, we used a nitrogen-limiting medium, mineral salts medium (MM) instead of rich nutrient medium such as LB and NR to promote the accumulation of PHA in the recombinant *C. necator* PHB¯4. This strain required nutrient-limiting conditions such as nitrogen, phosphorus, magnesium, sulphur or oxygen but at the same time with the supply of excess carbon source to initiate PHA biosynthesis [45,46].

### New source of PhaC from culture-independent studies

In this study, we have compiled the *phaC* sequences obtained from culture-independent studies [20-22,47] and compared their relationship with PhaCs from known PHA producers based on the phylogenetic tree. Bacterial genera with known PHA producers have been summarized by Verlinden *et al.* [10] and Koller *et al.* [44,48]. Interestingly, almost half of the PhaC sequences from culture-independent resources have not been examined for PHA productivity. A noticeable difference is the prevalence of Class I PhaCs of *Alphaproteobacteria* in seawater DNA with a higher proportion of uncharacterized PhaCs compared with other environmental DNA (activated sludges, soils and oil-contaminated soil) where the Class I PhaCs of *Betaproteobacteria* is the dominant group. This could be explained by the fact that *Alphaproteobacteria* is one of the most dominant bacterial groups found in pelagic seawater realms [3,4,29].

We can foresee that the number of uncharacterized PhaCs will increase in the near future due to the popularity of culture-independent approaches and bacterial whole genome sequencing. However, cautious interpretation is needed due to the existence of gene paralogs [49,50] where some bacteria harbor more than one copy of the *phaC* gene in their genome, such as in *C. necator*, *Bradyrhizobium japonicum*, *R. rubrum* [51-53]. In some cases, these paralogs show low sequence similarity or even belong to different PhaC classes [37,53]. Therefore, further evaluation of PhaC activity through either *in vitro* or *in vivo* approaches will be required, especially for PhaCs retrieved from culture-independent studies.

## Conclusions

A new functional PhaC that has high identity to that of *Marinobacter* sp. has been discovered and PHA accumulation was observed in recombinant *C. necator *PHBˉ4. Further studies are currently ongoing to characterize the full potential of this newly identified PHA synthase. Seawater and other environmental resources harbor a great diversity of *phaC* genes with unexplored PhaC activity. Functional evaluation of these *in silico*-based PhaCs via genome walking has provided new insights into the polymerizing ability of the enzyme from rarely cultured microorganisms.

## Methods

### Seawater sampling

**Figure 3:**
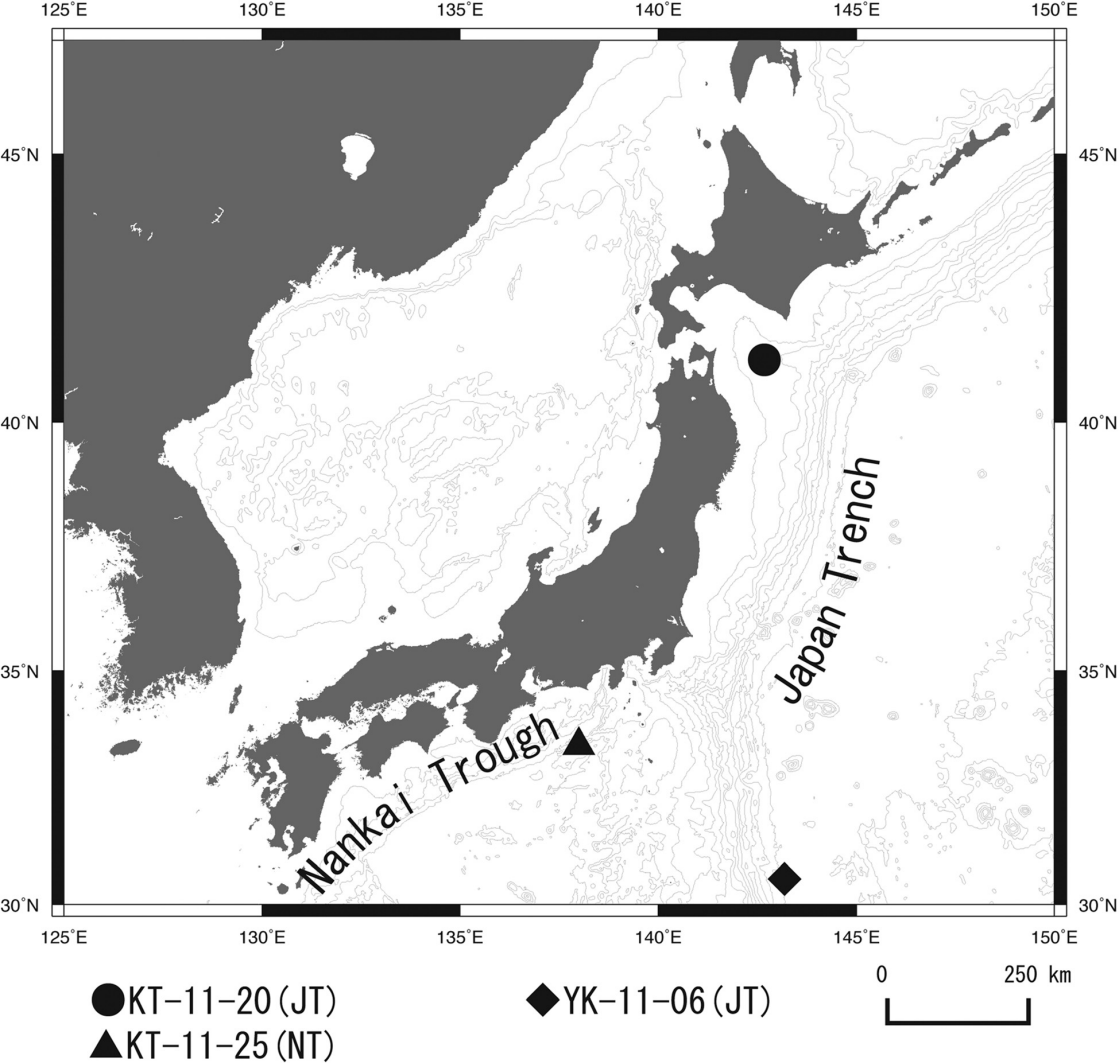
**Seawater sampling locations around the Japan Trench and Nankai Trough, North Pacific Ocean.**

The SHINKAI 6500 (JAMSTEC, Japan), a manned research submersible able to dive to a depth of 6500 meters and R/V Tansei maru (AORI, University of Tokyo/JAMSTEC) were used to collect the seawater samples. The samples were taken between August and October 2011 from three locations in the North Pacific Ocean around the Japan Trench and Nankai Trough at depths ranging from 24 m to 5373 m (Figure 3 and Table 3). The samples were immediately stored at −80°C.

**Table 3:** **GPS coordinates and description of the sampling sites**

**Sampling site name**	**Cruise name** ^**a**^	**GPS coordinates**	**Depth (meters)**	**Temperature (°C)**
**10**	KT-11-20 (JT)	41°14.4879′N	24	17.9
		142°59.4645′E		
**7**	KT-11-20 (JT)	41°14.4879′N	99	13.3
		142°59.4645′E		
**5**	KT-11-20 (JT)	41°14.4879′N	199	4.6
		142°59.4645′E		
**2**	KT-11-20 (JT)	41°14.4879′N	1000	2.9
		142°59.4645′E		
**1**	KT-11-20 (JT)	41°14.4879′N	1913	2.0
		142°59.4645′E		
**3000**	KT-11-25 (NT)	33°4.999′N	3000	1.6
		137°36.992′E		
**YK**	YK-11-06 (JT)	30°8.9596′N	5373	1.5
		143°34.9867′E		

^a^JT = Japan Trench; NT = Nankai Trough.

### Total DNA extraction and whole genome amplification (WGA)

Seven seawater samples were selected for this work. Approximately 900 mL of each sample was defrosted and filtered through 0.22 μm pore-sized Sterivex-GP cartridge filters (Millipore, USA) [54]. After filtering, the cartridges were opened and the filter membranes were carefully removed with sterilized forceps and transferred into Lysing Matrix E tubes, which come with the FastDNA™ 2 mL SPIN Kit for Soil (MP Biomedicals, USA). All of the proceeding steps were carried out under aseptic conditions. Total DNA extraction was carried out according to the manufacturer’s protocol with slight modifications including the DNA extraction buffer composition, the addition of lysozyme and Proteinase K treatments, and a hot-lysis incubation at 65°C as described in Zhou *et al.* [55]. Whole genome amplification (WGA) was performed using the REPLI-g Mini Kit (QIAGEN, Germany) to increase the amounts of DNA to sufficient levels (>1 μg) for subsequent experimental analyses.

### Screening of PHA synthase (*phaC*) genes from seawater WGA DNA

Degenerate primers G-D and G-1R (Additional file 6: Table S1) specifically targeting Class I and II PHA synthases [12] were used to amplify the *phaC*, *phaC1 and phaC2* genes from the WGA seawater DNA. The PCRs contained 1× *Ex Taq* buffer, 1.5 mM MgCl_2_, 200 μM dNTPs, 0.25 μM of each primer, 1% DMSO and 1 U of *Ex Taq* (TaKaRa, Japan). The PCR conditions were as follows: 94°C for 3 min; 30 cycles of 94°C for 30 s, 55°C for 30 s, and 72°C for 50 s; a final step at 72°C for 7 min. PCR products were run on an agarose gel and those within the range of 500 to 600 bp were excised from the gel, purified, and inserted into the pCR®4-TOPO® vector (Invitrogen, USA). The vectors were transformed into *E. coli* DH5α (TaKaRa, Japan) and selected on Lysogeny-Broth (LB) plates containing 50 μgmL^−1^ kanamycin. Plasmids from the positive clones were isolated using PowerPrep™ Express Plasmid Purification Kits (ORIGEN, USA) and DNA sequencing was carried out at the RIKEN Center for Sustainable Resource Science (CSRS), Yokohama, Japan.

### Sequence analyses

The MegAlign tool (DNASTAR, USA) was used to calculate nucleotide sequence similarity. Clones showing ≥90% identity in their nucleotide sequence were categorized as part of the same genetic group. Sequence similarity searches were conducted using BLASTX 2.2.30 against the GenBank non-redundant protein sequences database [56]. The ExPASy translation tool [57] was used to translate and predict the correct reading frames for the nucleotide sequences. Partial PhaC sequences were aligned using the MUSCLE algorithm [58] of the BioEdit program [59]. Protein sequences were also checked for the presence of the conserved catalytic lipase-box (G-X-[S/C]-X-G) [9]. Protein neighbor-joining (NJ) phylogenetic trees were constructed using MEGA5 [60] with MUSCLE alignment and 1000 bootstrap tests.

### Genome walking with IAN-PCR

Genome walking with inverse affinity nested PCR (IAN-PCR) was carried out to obtain the full-length *phaC* DNA sequences [61]. WGA seawater DNA was digested with FastDigest *Eco*RI (Fermentas, Lithuania) and self-ligated using a DNA Ligation Kit Ver1.2 (TaKaRa, Japan). For each genetic group, two sets of primers (for two rounds of amplification) (Additional file 6: Table S1) were designed based on the partial *phaC* sequences obtained in the previous step. The first round of amplification (inverse affinity PCR) included a 5′-biotinylated primer that enabled the product to be purified using the KiloBase Binder Kit (Invitrogen, USA) and serve as a template for the second round of amplification (nested PCR). DNA sequences were determined by performing the same cloning and sequencing steps described in the previous section. Full-length *phaC* sequences were predicted *in silico* by assembling the genome walking DNA fragments using the SeqMan tool (DNASTAR, USA).

### Nucleotides sequence accession numbers

The nucleotide sequences were submitted to the GenBank nucleotide database. Partial *phaC* sequences were assigned accession numbers [GenBank: KF911019-KF911073] as were the complete coding sequences (CDS) of the *phaC* genes [GenBank: KF911074-KF911076].

### Heterologous expression of PHA synthases (*phaC*) in *C. necator* PHBˉ4

The ribosome binding sequence (RBS) upstream of the start codon for each *phaC* gene was optimized for *C. necator* via the RBS calculator [62]. The gene constructs were assembled in the following order with appropriate restriction sites (Additional file 6: Table S1): *phaC1* promoter from *C. necator,* optimized RBS, and complete CDS of the *phaC* genes. After restriction digestion, the gene constructs were inserted into plasmid pBBR1MCS-2 [63] in reverse orientation to the *lac* promoter to ensure that expression was solely controlled by the *C. necator phaC1* promoter. Bacterial conjugation of *C. necator* PHBˉ4 with *E. coli* S17-1 [64] harboring a broad host range vector (pBBR1MCS-2 derivatives) was conducted as described by Friedrich *et al.* [65]. The presence of the recombinant PHA synthase in recombinant *C. necator* PHB¯4 was confirmed with direct PCR on the bacterial DNA. Wild-type *C. necator* H16 and PHB-negative mutant *C. necator* PHBˉ4 were used as the positive and negative controls for PHA production, respectively (Additional file 7: Table S2). All bacterial strains were pre-cultured at 30°C, 200 rpm for 6 h in 50 mL of Nutrient Rich (NR) medium (300 μgmL^−1^ kanamycin). After culturing, 3% (v/v) of the inoculum was transferred into 50 mL of mineral salts medium (MM) (300 μgmL^−1^ kanamycin) with 20 gL^−1^ of fructose added as the sole carbon source, and incubated at 30°C, at 200 rpm for 48 h.

### PHA content quantification

The cells were harvested by centrifugation (8,000 *g* for 10 min), washed once with distilled water and kept at −20°C for 24 h before lyophilization. The dry cell weight of each culture was gravimetrically determined and approximately 20 mg of lyophilized cells were subjected to methanolysis in the presence of 15% (v/v) sulfuric acid and 85% (v/v) methanol at 100°C for 140 min [66]. The PHA content was determined by gas chromatography (GC) using the Shimadzu GC-2010 system equipped with column SPB-1 (Supelco, USA). The column temperature was initiated at 70°C and then increased to 280°C in a continuous step of 10°C/min. The PHA content and composition were quantified with caprylic acid methyl ester as an internal standard.

## Additional files

Additional file 1: Figure S2. Protein neighbor-joining phylogenetic tree of PHA synthases obtained from WGA seawater DNA. Solid diamonds indicate the consensus PhaC sequence within the same genetic group (nucleotide sequence identity >90%). White diamonds indicate PhaC sequences with complete CDSs. The scale represents the number of amino acid substitutions per site. Bootstrapping values less than 50 are not shown in the tree. Additional file 2: Figure S1. Multiple sequence alignment of the partial PhaC proteins obtained from WGA seawater DNA. A MSA was created with Class I and II PhaCs from known PHA producers using MUSCLE. There are three PhaC genetic groups comprised solely of pseudogenes. The location of the putative lipase box (G-X-[S/C]-X-G-G) is outlined. Dark colored columns represent completely conserved amino acids. Abbreviation: A. = *Aeromonas*; B. = *Bradyrhizobium*; C. = *Cupriavidus*; P. = *Pseudomonas.*
Additional file 3: Table S3. Genetic groups and closest organism matches of PHA synthases for the partial *phaC* genes. Additional file 4: Table S4. Accession numbers for all the PHA synthases used in the protein neighbor-joining phylogenetic tree. Additional file 5: Figure S3. Multiple sequence alignment of three putative complete PhaC CDSs with known functional PHA synthases. A MSA was created with Class I and II PhaCs from known PHA producers using MUSCLE. The location of the putative lipase box (G-X-[S/C]-X-G-G) is outlined. Eight highly conserved amino acid residues are indicated by arrows. Dark colored columns represent completely conserved amino acids. Abbreviation: A. = *Aeromonas*; B. = *Bradyrhizobium*; C. = *Cupriavidus*; P. = *Pseudomonas.*
Additional file 6: Table S1. List of primers used in this study. Additional file 7: Table S2. List of plasmids and strains used in this study.
