# Supplementary figures and images for: Whole genome amplification approach reveals novel polyhydroxyalkanoate synthases (PhaCs) from Japan Trench and Nankai Trough seawater
Source: BMC Microbiol. 2014 Dec 24;14:318. doi: 10.1186/s12866-014-0318-z (PMC4326521; doi:10.1186/s12866-014-0318-z)

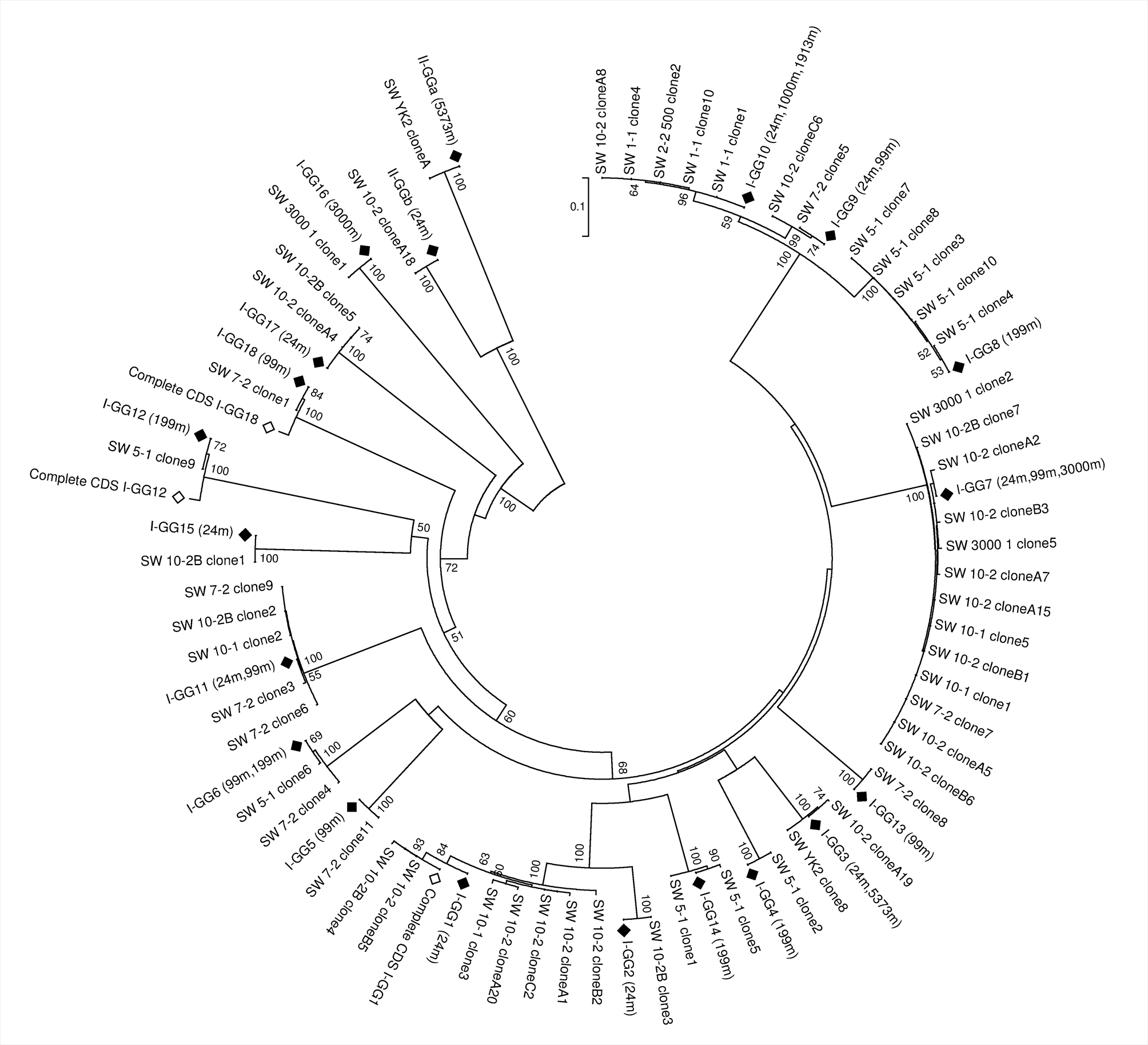

Supplement: Additional file 1: Figure S2. — Protein neighbor-joining phylogenetic tree of PHA synthases obtained from WGA seawater DNA. Solid diamonds indicate the consensus PhaC sequence within the same genetic group (nucleotide sequence identity >90%). White diamonds indicate PhaC sequences with complete CDSs. The scale represents the number of amino acid substitutions per site. Bootstrapping values less than 50 are not shown in the tree. [file 12866_2014_318_MOESM1_ESM.tiff]

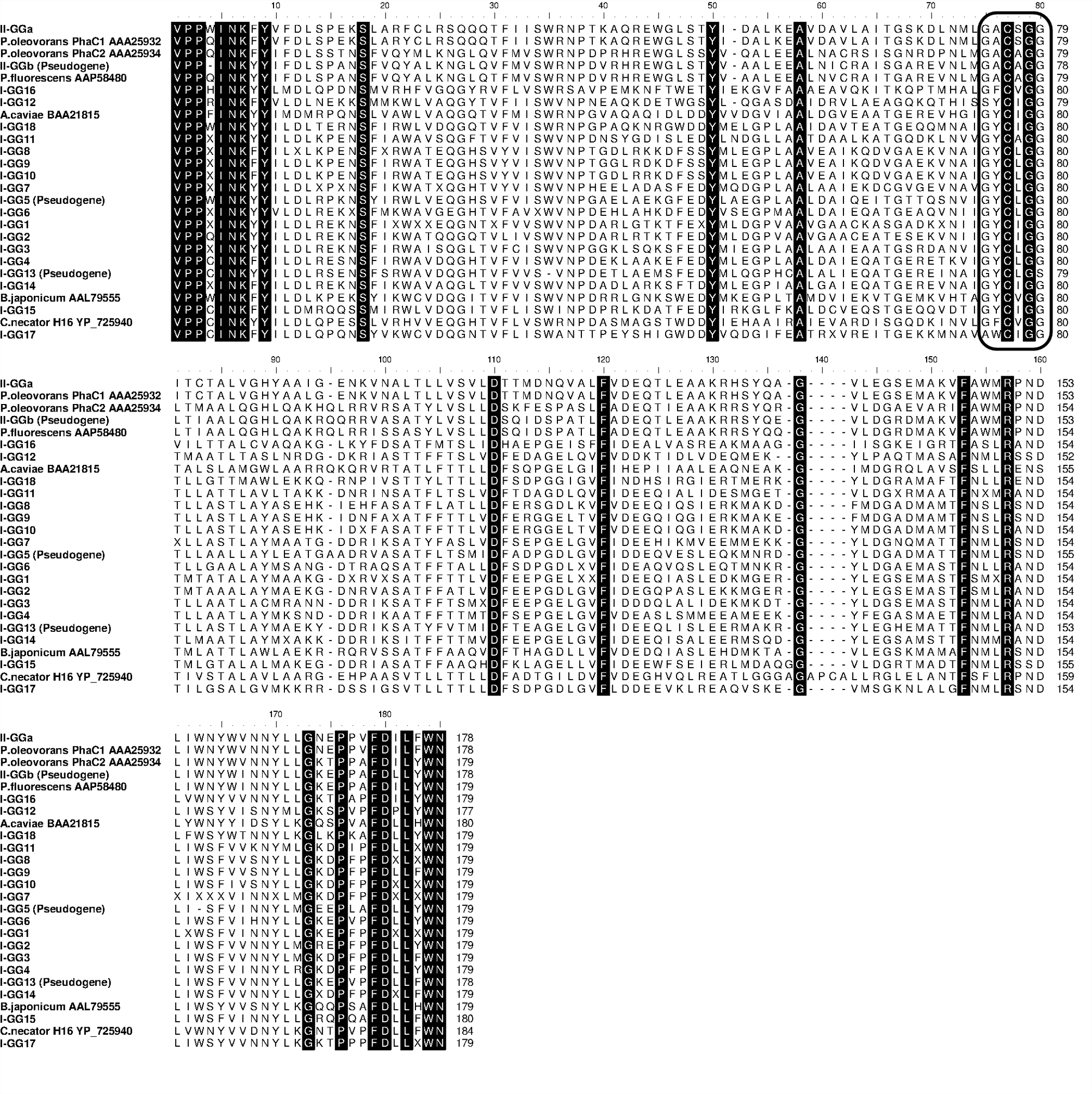

Supplement: Additional file 2: Figure S1. — Multiple sequence alignment of the partial PhaC proteins obtained from WGA seawater DNA. A MSA was created with Class I and II PhaCs from known PHA producers using MUSCLE. There are three PhaC genetic groups comprised solely of pseudogenes. The location of the putative lipase box (G-X-[S/C]-X-G-G) is outlined. Dark colored columns represent completely conserved amino acids. Abbreviation: A. = Aeromonas; B. = Bradyrhizobium; C. = Cupriavidus; P. = Pseudomonas. [file 12866_2014_318_MOESM2_ESM.tiff]

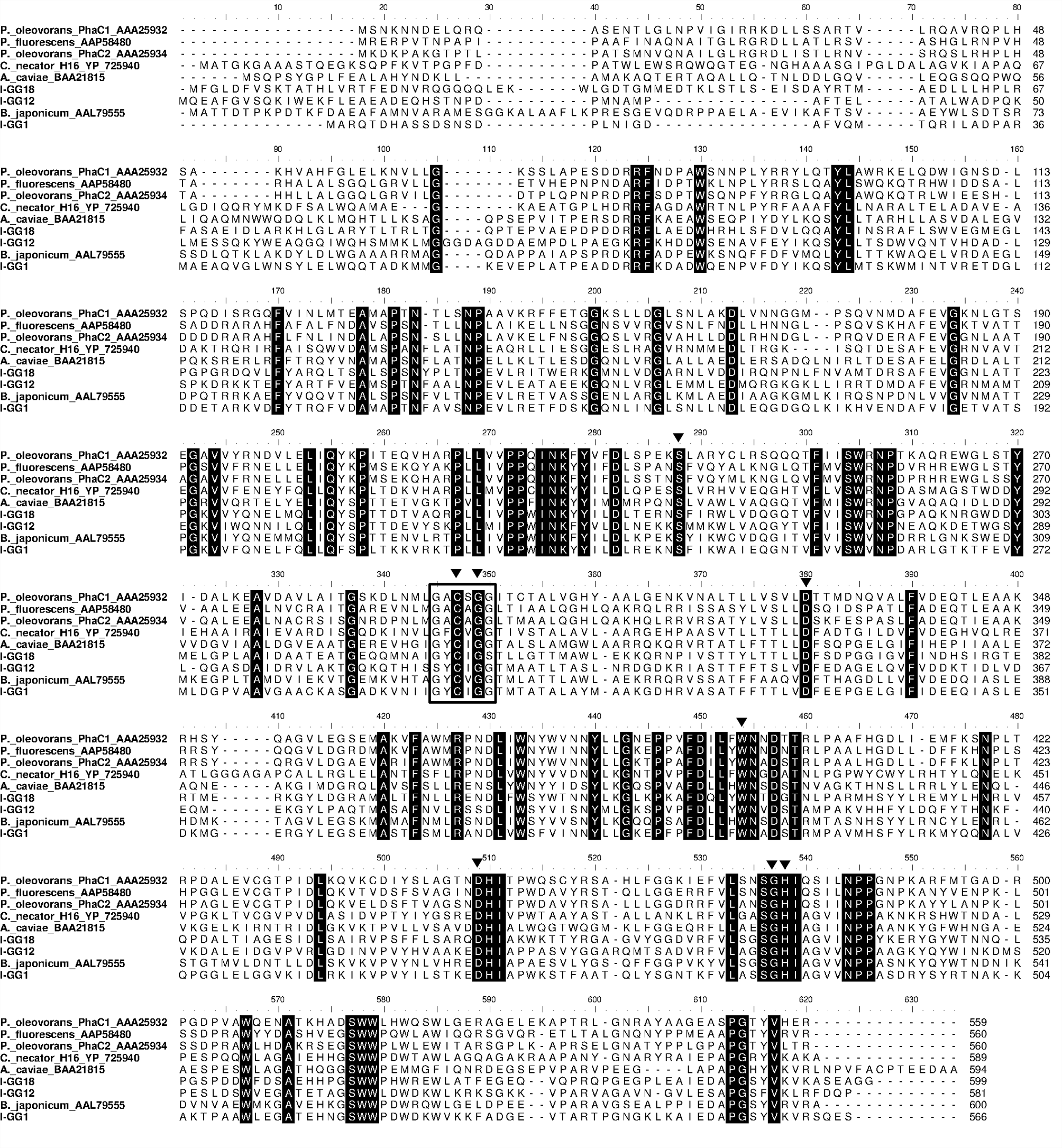

Supplement: Additional file 5: Figure S3. — Multiple sequence alignment of three putative complete PhaC CDSs with known functional PHA synthases. A MSA was created with Class I and II PhaCs from known PHA producers using MUSCLE. The location of the putative lipase box (G-X-[S/C]-X-G-G) is outlined. Eight highly conserved amino acid residues are indicated by arrows. Dark colored columns represent completely conserved amino acids. Abbreviation: A. = Aeromonas; B. = Bradyrhizobium; C. = Cupriavidus; P. = Pseudomonas. [file 12866_2014_318_MOESM5_ESM.tiff]
