# Supplementary material for: Whole genome amplification approach reveals novel polyhydroxyalkanoate synthases (PhaCs) from Japan Trench and Nankai Trough seawater
Source: BMC Microbiol. 2014 Dec 24;14:318. doi: 10.1186/s12866-014-0318-z (PMC4326521; doi:10.1186/s12866-014-0318-z)
Supplement: Additional file 3: Table S3. — Genetic groups and closest organism matches of PHA synthases for the partial phaC genes. [file 12866_2014_318_MOESM3_ESM.docx]

**Table S3:** Genetic groups and closest organism matches of PHA synthase for the partial *phaC* genes.

| Genetic group^a^ | Depth (meters) | No. of clones | Closest organism match^b^ | PhaC class | Identity (%) |
| --- | --- | --- | --- | --- | --- |
| I-GG1 | 24 | 7 | *Nisaea denitrificans* (WP_028465014) | I | 71-74 |
| I-GG2 | 24 | 1 | *Nisaea denitrificans* (WP_028465014) | I | 70 |
| I-GG3 | 24 | 1 | *Nisaea denitrificans* (WP_028465014) | I | 80 |
| I-GG3 | 5373 | 1 | *Nisaea denitrificans* (WP_028465014) | I | 80 |
| I-GG4 | 199 | 1 | *Azospirillum halopraeferens* (WP_029009957) | I | 78 |
| I-GG5* | 99 | 1 | *Rhodospirillum rubrum* (WP_011390166) | I | 71 |
| I-GG6 | 99 | 1 | *Nisaea denitrificans* (WP_028465014) | I | 72 |
| I-GG6 | 199 | 1 | *Nisaea denitrificans* (WP_028465014) | I | 72 |
| I-GG7 | 24 | 1 | *Elioraea tepidiphila* (WP_019014986) | I | 71 |
| I-GG7 | 24 | 9 | *Rhodospirillum centenum* (WP_012566500) | I | 69-72 |
| I-GG7 | 99 | 1 | *Rhodospirillum centenum* (WP_012566500) | I | 70 |
| I-GG7 | 3000 | 2 | *Rhodospirillum centenum* (WP_012566500) | I | 70-72 |
| I-GG8 | 199 | 5 | *Caenispirillum salinarum* (WP_009542208) | I | 67-68 |
| I-GG9 | 24 | 1 | *Thalassospira profundimaris* (WP_008890930) | I | 68 |
| I-GG9 | 99 | 1 | *Thalassospira profundimaris* (WP_008890930) | I | 66 |
| I-GG10 | 24 | 1 | *Thalassospira profundimaris* (WP_008890930) | I | 68 |
| I-GG10 | 1000 | 1 | *Thalassospira profundimaris* (WP_008890930) | I | 68 |
| I-GG10 | 1913 | 3 | *Thalassospira profundimaris* (WP_008890930) | I | 68 |
| I-GG11 | 24 | 2 | *Nisaea denitrificans* (WP_028465014) | I | 66-67 |
| I-GG11 | 99 | 3 | *Nisaea denitrificans* (WP_028465014) | I | 66-68 |
| I-GG12 | 199 | 1 | *Meganema perideroedes* (WP_018631852) | I | 57 |
| I-GG13* | 99 | 1 | *Acetobacteraceae bacterium* AT-5844 (WP_007438224) | I | 79 |
| I-GG14 | 199 | 2 | *Thalassobaculum salexigens* (WP_028794541) | I | 83 |
| I-GG15 | 24 | 1 | *Oceanicaulis* sp. HTCC2633 (WP_009800822) | I | 81 |
| I-GG16 | 3000 | 1 | *Chromobacterium* sp. USM2 (ADL_70203) | I | 98 |
| I-GG17 | 24 | 2 | *gamma proteobacterium* HIMB30 (WP_010547143) | I | 92 |
| I-GG18 | 99 | 1 | *Marinobacter sp.* EN3 (WP_023010452) | I | 99 |
| II-GGa | 5373 | 1 | *Pseudomonas putida* (YP_001270181) | II | 99 |
| II-GGb* | 24 | 1 | *Pseudomonas fluorescens* (AIG04370) | II | 98 |

^a^Genetic groups were defined with a cutoff of ≥90% identity in nucleotide sequence

^b^BLASTX against the GenBank non-redundant protein sequences database (nr), date: 21st September 2014

*Genetic group comprised solely of pseudogenes
