# Supplementary material for: Whole genome amplification approach reveals novel polyhydroxyalkanoate synthases (PhaCs) from Japan Trench and Nankai Trough seawater
Source: BMC Microbiol. 2014 Dec 24;14:318. doi: 10.1186/s12866-014-0318-z (PMC4326521; doi:10.1186/s12866-014-0318-z)
Supplement: Additional file 6: Table S1. — List of primers used in this study. [file 12866_2014_318_MOESM6_ESM.docx]

**Table S1:** List of primers used in this study.

| Primer name^a^ | Nucleotide sequence (5’ to 3’) | Source or reference |
| --- | --- | --- |
| Partial *phaC* primers |  |  |
| G-D | GTG CCG CCS YRS ATC AAC AAG T | [12] |
| G-1R | GTT CCA GWA CAG SAK RTC GAA | [12] |
| IAN-PCR primers (for metagenome walking) |  |  |
| B-GG1-IAF | GTG TTG CCC TGT TCT GTC ACC CAT TTG | This study |
| GG1-IAR | GAA CAG ATC GCC AGC CTC GAA GAC AAG | This study |
| GG1-NF | TTT CGC GCA GGT CGA GGA TG | This study |
| GG1-NR | AAC AAC TAT CTG CTC GGC AAG G | This study |
| B-GG12-IAF | CAT CCC GAT TAA GGC TTG CGG TCA G | This study |
| GG12-IAR | CGA ACT GCA AGT GTT TGT TGA CGA C | This study |
| GG12-NF | GCT ACC AGC CAC TTC ATC ATC G | This study |
| GG12-NR | GGC AAA TCA CCA GTT CCG TTC G | This study |
| B-GG18-IAF | TTC ATC TGT TGC TCG CCG GTC GCT TC | This study |
| GG18-IAR | CGG CGG CAT CGG CGT CTT CAT TAA C | This study |
| GG18-NF | TCT ACC AGC CAG CGA ATG AAG G | This study |
| GG18-NR | CGA TCT GTT CTG GTC CTA CTG G | This study |
| *C. necator phaC1* promoter primers |  |  |
| *Sac*I_Cn_Prom-f | AGT GAGCTC AGT ACC TTG CCG ACA TCT ATG C | This study |
| *Bam*HI_Cn_Prom-f | AGT GGATCC AGT ACC TTG CCG ACA TCT ATG C | This study |
| *Hin*dIII_Cn_Prom-r | AGC AAGCTT GTC TCT CTG CCG TCA CTA TTC G | This study |
| Complete CDS *phaC* primers |  |  |
| *Hin*dIII_RBS_GG1-f | AGT AAGCTT **AAGGGGGTATCA** ATG GCC CGC CAG ACG GAT CAT G | This study |
| *Apa*I_GG1-r | ATT GGGCCC AAT TCG TTC AGC TTT CCT GCG AGC | This study |
| *Hin*dIII_RBS_GG12-f | AGC AAGCTT **AGGACCAGTAAT** ATG CAG GAG GCT TTC GG | This study |
| *Apa*I_GG12-r | ATT GGGCCC TCT TTG GCA CGC TAA CTG AAC G | This study |
| *Hin*dIII_RBS_GG18-f | AGC AAGCTT **AAGGAGGGAAAC** ATG TTC GGT CTG GAT TTC GTG AG | This study |
| *Sal*I_GG18-r | AGT GTCGAC GTT GTT CTT GGT CTG TCT GTT GC | This study |

^a^All primers were synthesized by Hokkaido System Science Co. Ltd. (Japan) and Integrated DNA Technologies Inc. (USA). Restriction enzyme digestion sites are underlined. Bold letters represent the optimized ribosome binding sequence (RBS).
