# Supplementary material for: Whole genome amplification approach reveals novel polyhydroxyalkanoate synthases (PhaCs) from Japan Trench and Nankai Trough seawater
Source: BMC Microbiol. 2014 Dec 24;14:318. doi: 10.1186/s12866-014-0318-z (PMC4326521; doi:10.1186/s12866-014-0318-z)
Supplement: Additional file 7: Table S2. — List of plasmids and strains used in this study. [file 12866_2014_318_MOESM7_ESM.docx]

**Table S2:** List of plasmids and strains used in this study.

| Plasmid/ strain name | Relevant characteristics | Source or reference |
| --- | --- | --- |
| Plasmid |  |  |
| pCR®4-TOPO® | Km^r^, cloning vector | Invitrogen |
| pBBR1MCS-2 | Km^r^, *mob*, *lacZα*, broad-host-range cloning vector | [63] |
| pBBR1-Pro*_cn_*GG1 | pBBR1MCS-2 derivatives with *phaC1* promoter from *C. necator* and PhaC-CLS1-GG1 | This study |
| pBBR1-Pro*_cn_*GG12 | pBBR1MCS-2 derivatives with *phaC1* promoter from *C. necator* and PhaC-CLS1-GG12 | This study |
| pBBR1-Pro*_cn_*GG18 | pBBR1MCS-2 derivatives with *phaC1* promoter from *C. necator* and PhaC-CLS1-GG18 | This study |
| pBBR1-GG18 | pBBR1MCS-2 derivatives with PhaC-CLS1-GG18 | This study |
| Strain |  |  |
| *E. coli* DH5α | F^−^, φ 80d*lacZ*ΔM15, Δ(*lacZYA*-*argF*)U169, *deoR*, *recA1*, *endA1*, *hsdR17*, *phoA*, *supE44*, λ^−^, *thi*-1, *gyrA96*, *relA1* | Takara |
| *E. coli* S17-1 | 294 derivative RP4-2Tc::Mu-Km::Tn7 chromosomally integrated | [64] |
| *C. necator* H16 | Wild type | ATCC 17699 |
| *C. necator* PHBˉ4 | PHB-negative mutant of H16 | DSM 541 |
